# Editorial: Role of mitochondria in post-traumatic stress disorder (PTSD)

**DOI:** 10.3389/fphys.2023.1341204

**Published:** 2023-12-14

**Authors:** Graziano Pinna, Hanna Kmita, Volodymyr I. Lushchak

**Affiliations:** ^1^ Psychiatric Institute (SPHPI), Chicago, IL, United States; ^2^ UI Center on Depression and Resilience (UICDR), Chicago, IL, United States; ^3^ Center for Alcohol Research in Epigenetics (CARE), Department of Psychiatry, College of Medicine, University of Illinois at Chicago, Chicago, IL, United States; ^4^ Department of Bioenergetics, Institute of Molecular Biology and Biotechnology, Faculty of Biology, Adam Mickiewicz University, Poznań, Poland; ^5^ Department of Biochemistry and Biotechnology, Vasyl Stefanyk Precarpathian National University, Ivano-Frankivs, Ukraine; ^6^ Research and Development University, Ivano-Frankivsk, Ukraine

**Keywords:** PTSD—posttraumatic stress disorder, oxidative stress, biomarkers for PTSD, PTSD treatments, mitochondria

Among psychiatric disorders, post-traumatic stress disorder (PTSD) remains one of the most under-diagnosed and under-treated (Raber et al., 2019). No specific treatments have as of today been developed; however, PTSD has a high prevalence in the population (Oakley et al., 2021). When untreated, PTSD often tends to deteriorate over time. PTSD is also highly comorbid with major depression, suicidality, substance use disorder, and traumatic brain injury (TBI), which makes it even more difficult to manage therapeutically (Mann and Marwaha, 2023).

PTSD symptoms develop after experiencing or witnessing a traumatic event and persist for over 1 month and may last for years (Merians et al., 2023). Trauma includes being victims of violence, terrorist attacks, participation in warzone, physical or psychological abuse, witnessing scenes of cruelty, surviving natural disasters, and being exposed to domestic abuse that can strongly affect children and adults (Ross et al., 2021). Main symptoms comprise re-experiencing and avoidance of trauma-related cues and hyperarousal (Raber et al., 2019). Nightmares and sleep disturbance, including insomnia occur often. Treatments, such as the selective serotonin reuptake inhibitors are the only approved drugs, but they improve symptoms in only a small portion of subjects, which strongly support the necessity to discover novel efficient drugs (Bandelow et al., 2023).

There are no established biomarkers to predict treatment response to drugs or improve diagnosis of PTSD (Pinna, 2018). Thus, investigation for clinically relevant, accurate, and reproducible biomarkers is highly prioritized. With advances in large-scale multi-omic investigations including genomic, proteomic, and metabolomic data, progress has been achieved, but have yet to be implemented into clinical practice. It is especially important to develop fast and noninvasive methods for PTSD diagnosis.

An imbalance in the hypothalamic-pituitary adrenal axis (HPA) (Szeszko et al., 2018) function and increased neuroinflammation (Hori and Kim, 2019) are regarded as main components of PTSD neurobiology. Mitochondrial dysfunction represents an emerging trend in PTSD research. Rodent models of PTSD focus on the relation of trauma-eliciting fear-related learning, avoidance, emotional and cognition deficits, and arousal behaviors to altered mitochondrial dysfunction in relevant neuronal pathways (Aspesi and Pinna, 2019). Thus, this Research Topic focuses on the emerging role of mitochondria in regulating mechanisms of PTSD pathophysiology, including oxidative stress and inflammation. A total of six contributions discusses new aspects of mitochondria contribution to PTSD and potential treatment approaches to alleviate PTSD.

Redox molecules are reactive species including free radicals, which are generated from the mitochondrial electron transport chain, necessary for sustaining life. While these reactive molecules are essential for life, when present in excess they may lead to “oxidative stress” that underlies several pathophysiological conditions. Reed and Case (Reed and Case) discuss biomarkers for PTSD focusing on redox biology. They provide a foundation of how redox processes may underline PTSD neurobiology and discuss redox biomarker studies related to PTSD. Directions for implementing the field with standardized, reproducible, and accurate redox assessments for diagnostic, prognostic, and therapeutic strategies of PTSD are provided.

Dmytriv et al. focus on mitochondria involvement in the development of neuroinflammation in PTSD etiopathology. They describe the neuroendocrine system differences under acute stress and PTSD pathological conditions and changes in the activity and expression of mitochondrial proteins affecting hormonal levels in PTSD pathophysiology. They detail how mitochondrial damage- and pathogen-associated molecular patterns (DAMPs/PAMPs) may trigger the development of proinflammatory processes. Finally, these authors discuss the possibility of treating PTSD-related inflammation by targeting mitochondria function to improve symptoms.

Kmita et al. report on mitochondrial dysfunction, which results from chronic oxidative stress that previous studies linked with alterations in neurotransmitter signaling and altered inflammatory response. They discuss the mechanisms by which mitochondrial dysfunction may underlie development of PTSD symptoms and how this may even lead to increased PTSD susceptibility. Importantly, they highlight therapeutic strategies to target oxidative stress and prevent/treat PTSD symptoms through mitochondrial-mediated, endogenous antioxidative mechanisms, including mitochondria-derived peptides and vesicles. The mitochondria-derived peptides are known from their antioxidant activity within mitochondria as well as from acting as paracrine and endocrine signaling molecules whereas mitochondria-derived vesicles emerge as an essential mitochondria quality control mechanism. These vesicles may be part of a cytoprotective mechanism consisting of the transferring of “repair packages” from one cell to another although the mechanism can be impaired by oxidative stress. Such mitochondria-derived peptides and vesicles can be used either as direct targets for therapies, or in concert with antioxidants and monoamine oxidase inhibitors.

Kaplan et al. focus on mitochondrial dysfunction within key brain regions with an approach integrating behavioral, neural, and cellular research in PTSD models, including fear conditioning, predator/social stress, chronic restraint stress, single prolonged stress, social isolation, chronic unpredictable stress, and early life stress models. Mitochondrial dysfunction including dysregulation of oxidative phosphorylation and other metabolic pathways such as beta-oxidation of fatty acids and the tricarboxylic acid cycle was observed in PTSD models. Neural reactive oxygen species (ROS) that damage mitochondrial DNA, proteins, and lipids are reported. Mitochondrial structure and biogenesis are reviewed in trauma models that affect neuroinflammatory responses, signal transduction, and regulate cell death. Antidepressants rescue these alterations, also improving stress-induced elements of brain mitochondrial dysfunction. These authors highlight mitochondrial mechanisms associated with PTSD-like behaviors among several accepted PTSD models and identify potential targets for the development of more effective treatment for PTSD.

Lushchak et al. report on existing data on PTSD causes and symptoms by highlighting the pivotal role of mitochondria and oxidative stress development. Excessive and/or prolonged exposure to traumatic experiences can cause irreversible mitochondrial damage leading to cell death. They suggest that data integration on mechanisms and function of the mitochondrial stress response will help to model more closely PTSD deficits important to obtain a comprehensive, universal, multifaceted, and effective strategy for managing PTSD.

Traumatic brain injuries (TBI), a condition highly comorbid with PTSD, can be aggravated by mitochondrial dysfunction including exponential ROS generation, changes in the mitochondrial inner membrane potential and altered mitochondrial dynamics. Shah and colleagues (Shah et al.) evaluate the effect of near-infrared (NIR) light exposure on gene expression in a *Drosophila* TBI model. NIR light interacts with cytochrome *c* oxidase (COX) of the electron transport chain to depolarize the mitochondrial inner membrane potential and attenuate ROS generation, as well as apoptosis. Inhibition of COX through NIR exposure resulted in downregulation of gene expression related to “nervous system development”, “neurogenesis,” “oxidation-reduction process,” and “intracellular transport” in a sex-dependent fashion.

This Research Topic dedicated to the mitochondria role in PTSD pathophysiology aims to attract interest on the emerging role of mitochondria targets both in biomarker and drug discovery and to fill the experimental gap in procedures involving animal models of PTSD (Aspesi and Pinna, 2019), to enhance translational research in this field with the hope that this will take us closer to a better understanding of affective research (Schiller et al., 2023). Lastly, elucidating the role of mitochondria in the pathophysiology of PTSD could herald new avenues for the prevention, diagnosis and treatment of this debilitating psychiatric disorder.

## References

[B1] Aspesi D. Pinna G. (2019). Animal models of post-traumatic stress disorder and novel treatment targets. Behav. Pharmacol. 30, 130–150. PMID: 30741728. 10.1097/FBP.0000000000000467 30741728

[B2] Bandelow B. Allgulander C. Baldwin D. S. Costa DLDC Denys D. Dilbaz N. (2023). World Federation of Societies of Biological Psychiatry (WFSBP) guidelines for treatment of anxiety, obsessive-compulsive and posttraumatic stress disorders - version 3. Part II: OCD and PTSD. World J. Biol. Psychiatry 24 (2), 118–134. Epub 2022 Jul 28. PMID: 35900217. 10.1080/15622975.2022.2086296 35900217

[B3] Hori H. Kim Y. (2019). Inflammation and post-traumatic stress disorder. Psychiatry Clin. Neurosci. 73 (4), 143–153. Epub 2019 Feb 21. PMID: 30653780. 10.1111/pcn.12820 30653780

[B4] Mann S. K. Marwaha R. (2023). Posttraumatic stress disorder. Treasure Island (FL): StatPearls. StatPearls Publishing; 2023 Jan–. PMID: 32644555. 32644555

[B5] Merians A. N. Spiller T. Harpaz-Rotem I. Krystal J. H. Pietrzak R. H. (2023). Post-traumatic stress disorder. Med. Clin. North Am. 107 (1), 85–99. Epub 2022 Oct 28. PMID: 36402502. 10.1016/j.mcna.2022.04.003 36402502

[B6] Oakley L. D. Kuo W. C. Kowalkowski J. A. Park W. (2021). Meta-analysis of cultural influences in trauma exposure and PTSD prevalence rates. J. Transcult. Nurs. 32 (4), 412–424. PMID: 33593236. 10.1177/1043659621993909 33593236

[B7] Pinna G. (2018). Biomarkers for PTSD at the interface of the endocannabinoid and neurosteroid Axis. Front. Neurosci. 12, 482. PMID: 30131663; PMCID: PMC6091574. 10.3389/fnins.2018.00482 30131663 PMC6091574

[B8] Raber J. Arzy S. Bertolus J. B. Depue B. Haas H. E. Hofmann S. G. (2019). Current understanding of fear learning and memory in humans and animal models and the value of a linguistic approach for analyzing fear learning and memory in humans. Neurosci. Biobehav Rev. 105, 136–177. Epub 2019 Apr 7. PMID: 30970272. 10.1016/j.neubiorev.2019.03.015 30970272

[B9] Ross S. L. Sharma-Patel K. Brown E. J. Huntt J. S. Chaplin W. F. (2021). Complex trauma and Trauma-Focused Cognitive-Behavioral Therapy: how do trauma chronicity and PTSD presentation affect treatment outcome? Child. Abuse Negl. 111, 104734. Epub 2020 Nov 5. PMID: 33162104. 10.1016/j.chiabu.2020.104734 33162104

[B10] Schiller D. Yu A. N. C. Alia-Klein N. Becker S. Cromwell H. C. Dolcos F. (2023). The human affectome. Neurosci. Biobehav Rev., 105450. Epub ahead of print. PMID: 37925091. 10.1016/j.neubiorev.2023.105450 37925091 PMC11003721

[B11] Szeszko P. R. Lehrner A. Yehuda R. (2018). Glucocorticoids and hippocampal structure and function in PTSD. Harv Rev. Psychiatry 26 (3), 142–157. PMID: 29734228. 10.1097/HRP.0000000000000188 29734228

